# Novel MRI‐Guided Ultrasound Equations for Whole‐Body Muscle Mass in Caucasian Adults

**DOI:** 10.1002/jcsm.70242

**Published:** 2026-03-19

**Authors:** Jona Van den Broeck, Hubert Raeymaekers, Erik Cattrysse, Savanah Héréus, Aldo Scafoglieri

**Affiliations:** ^1^ Experimental Anatomy Research Group, Department of Physiotherapy and Human Anatomy, Faculty of Physical Education and Physiotherapy Vrije Universiteit Brussel Brussels Belgium; ^2^ Department of Radiology University Hospital Brussels Brussels Belgium

**Keywords:** equation, muscle mass, ultrasound, whole‐body

## Abstract

**Background:**

Muscle mass is a critical indicator of health and functionality, yet its accurate measurement remains challenging. Ultrasound offers a promising alternative, providing cost‐effective, non‐invasive assessments of surrogates of muscle mass: muscle thickness and cross‐sectional area (CSA). This study aims to develop and validate ultrasound‐derived equations for estimating whole‐body muscle mass in healthy Caucasian adults using magnetic resonance imaging (MRI) as the reference standard, incorporating CSA measurements to enhance the accuracy and applicability of these equations.

**Methods:**

We enrolled 211 healthy Caucasian adults (age: 42.0 years [29.0–58.0], 52% female) for whole‐body MRI and ultrasound examinations, assessing eight muscle thicknesses and seven CSAs across the right arm, trunk and leg. The sample was divided into a development group (two‐thirds) and a cross‐validation group (one‐third). Stepwise multiple regression established ultrasound equations in the development group and cross‐validation group. After successful cross‐validation, the full sample was used to create the most accurate and most practical equations.

**Results:**

The most accurate equation for estimating whole‐body muscle mass included ultrasound muscle thickness measurements of the forearm extensor, rectus abdominis, rectus femoris, biceps femoris and tibialis anterior muscles, CSA measurements of the triceps brachii and tibialis anterior muscles and sex, weight and BMI. This model achieved an adjusted *R* ^2^ of 0.942 and a standard error of estimate (SEE) of 1.7 kg. A more practical equation, requiring fewer measurements, focused on ultrasound muscle thickness and CSA of select arm, abdominal and leg muscles, combined with sex and height. This simplified model showed an adjusted *R* ^2^ of 0.927 and an SEE of 2.0 kg, offering a good balance between accuracy and measurement burden.

**Conclusions:**

The equations developed in this study enable accurate estimation of whole‐body muscle mass in a Caucasian population using ultrasound. A practical, time‐efficient equation with fewer variables is available alongside a more detailed equation that provides higher accuracy. Importantly, models that combine muscle thickness and CSA measurements demonstrated improved prediction accuracy (higher adjusted *R* ^2^ and lower SEE) compared to those using muscle thickness alone, supporting the inclusion of CSA in future applications.

## 1 Introduction

Muscle mass represents a fundamental component of overall health and functionality [1]. Extensive research has underscored its pivotal role in determining physical performance, metabolic health and even longevity [1]. Low muscle mass has been implicated in a spectrum of adverse outcomes, including physical impairment, increased risk of falls and fractures, diminished survival rates and exacerbated disease progression [2, 3, 4]. Additionally, conditions such as sarcopenia, cachexia, frailty and malnutrition are closely linked with reduced muscle mass [1, 5], further emphasizing its clinical significance.

Despite its importance, accurately quantifying muscle mass remains a challenge, primarily due to the limitations associated with conventional measurement techniques. Magnetic resonance imaging (MRI) and computed tomography (CT) are considered gold standards for assessing muscle mass, offering detailed anatomical information [5, 6, 7, 8, 9]. However, these modalities are hindered by their high cost, lack of portability and impracticality for large‐scale screening efforts [5, 6, 7, 9, 10]. Moreover, MRI is susceptible to motion artefacts, whereas CT exposes patients to ionizing radiation, posing inherent risks [9, 10].

In response to the limitations associated with traditional methods of muscle mass assessment, alternative modalities such as bioelectrical impedance analysis (BIA) and ultrasound have garnered attention for their accessibility and cost‐effectiveness [5, 11, 12]. Among these alternatives, ultrasound emerges as a particularly promising tool, offering non‐invasive, real‐time imaging of muscle architecture [2, 13]. Moreover, ultrasound measurements, including muscle thickness (MT) and cross‐sectional area (CSA), serve as essential inputs for predicting individual muscle mass [5]. Extensive research has demonstrated the validity and reliability of ultrasound in assessing muscle characteristics, including MT and CSA [13, 14, 15, 16, 17, 18]. Furthermore, reviews have highlighted the efficacy of ultrasound‐derived equations in estimating muscle mass, with studies validating their accuracy against standard techniques such as dual‐energy x‐ray absorptiometry (DXA) [19]. Current equations for estimating muscle mass have notable limitations [19]. Primarily, many equations rely on lean mass derived from DXA, which may not accurately represent actual muscle mass due to the inclusion of non‐muscle tissues [20]. Moreover, MRI‐based equations have exclusively been developed in Asian samples [8, 21], raising questions about their generalizability across diverse ethnicities [22]. There are currently no MRI‐based equations specifically derived for Caucasian populations, highlighting a significant gap in the research. Another constraint lies in ultrasound‐derived equations, which have thus far been developed solely based on MT measurements [19]. However, the emergence of extended‐field‐of‐view ultrasound technology enables the reliable measurement of muscle CSA [19]. Muscle CSA, measured with ultrasound, has been found to be valid compared to a CSA measurement on MRI [23] and also has a good correlation with muscle strength [24]. Yet, it remains unclear whether integrating CSA into these equations could enhance their usability.

In light of these considerations, this study aims to address the existing gaps in muscle mass assessment by establishing ultrasound‐derived equations for whole‐body muscle mass (WBMM) estimation in healthy Caucasian adults. By leveraging MRI as the reference standard, we aimed to develop robust and validated equations that can enhance the accuracy and reliability of muscle mass assessment in clinical and research settings. Finally, this study aims to investigate the potential impact of incorporating CSA measurements on the accuracy and applicability of muscle mass estimation equations.

## 2 Methods

### 2.1 Participants

Two hundred eleven volunteers participated in this study, contributing data from March 2022 to July 2023. Inclusion criteria required participants to be 18 years or older, of Caucasian ethnicity and in good general health. Exclusion criteria encompassed individuals with neurological or muscular disorders, recent surgeries within 16 weeks preceding examination, pregnant women or those who had given birth within the same timeframe. Additionally, MRI‐specific exclusion criteria were applied, including the presence of prostheses, big tattoos and metal implants.

Prior to the study's commencement, participants were instructed to abstain from engaging in physical activities on the assessment day. Upon initiation, participants received a comprehensive briefing on the research objectives, provided informed consent and completed a questionnaire detailing basic demographics such as age, sex, height and weight. The study protocol received approval from the ethics committee (B.U.N. 1432020000335). All participants underwent MRI and ultrasound examinations on the same day.

### 2.2 MRI Assessment and Analysis

MRI examinations were conducted using a GE Premier 3‐T scanner, employing a T1 Fast spin echo (FSE) acquisition with the following parameters: 1 cm slice thickness, 50 cm field of view, 256^2^ matrix (2 × 2 mm^2^ in‐plane resolution), 8 stacks with 24 slices per stack and 4 slices overlap, 600 ms repetition time (TR), 6.8 ms echo time (TE), covering a total distance of 170 cm. The MRI protocol took approximately 25 min to complete, and the scan quality was maximized using a head coil and two coil blankets; one placed over the participant's trunk and another over the legs.

The WBMM was measured using MRI, assessing both the left and right sides. The participants were positioned supine, with their hands placed around their abdomen. Positioning their arms in this manner ensured they remained within the field of view. Despite this arm positioning, achieving full visibility of the trunk and arms simultaneously on the scan was not feasible for all participants due to variations in body width. For wider participants, scanning was limited to the right side, with participant positioning adjusted within the MRI scanner to guarantee full visibility of at least the right half of the body. This choice was made to facilitate a comprehensive assessment and ensure comparability with ultrasound measurements, which were exclusively conducted on the right side. Contours were drawn exclusively on the right side and doubled to calculate WBMM. In cases where the abdomen was too wide, causing the arms resting on the abdomen to fall outside the field of view, the participant was excluded. For participants with full visibility of both body sides, muscle contours were delineated for the right and left sides. To clearly distinguish between right and left muscle mass on MRI, anterior anatomical landmarks were used in the following order from superior to inferior: the tracheal midline, sternum, linea alba, and symphysis pubis. Posteriorly, the separation was made using the spinous processes of the vertebral column as the dividing line.

**FIGURE 1:**
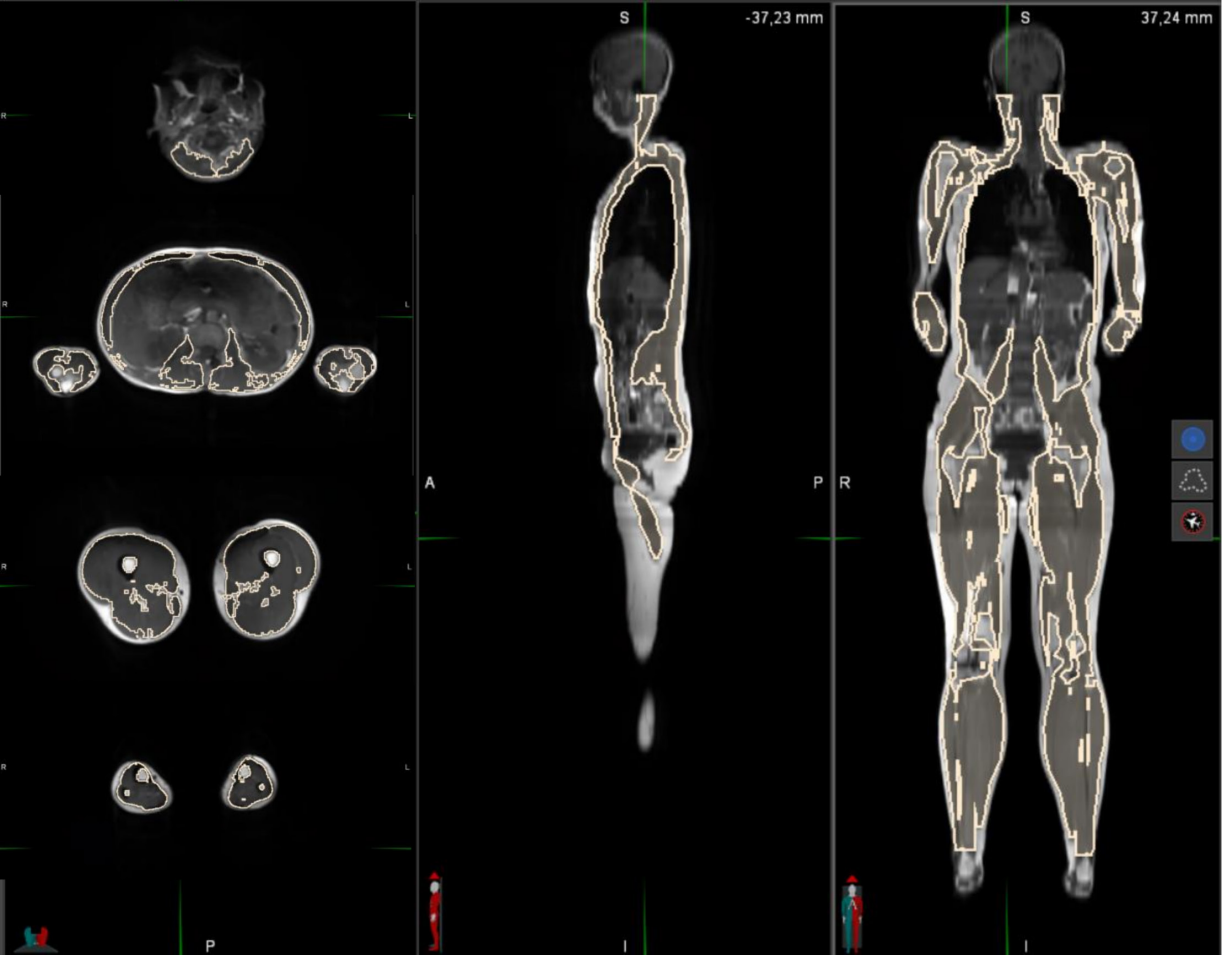
Contoured muscles on MRI. Female participant, 28 years old, height: 1.65 m, weight: 58 kg, muscle mass: 21.2 kg.

The images were imported into MIM Software Inc.'s 64‐bit version 7.3.2 [25] for analysis. Muscle contours were delineated semi‐manually using the 2D brush tool, starting from the level of the first cervical vertebra down to the malleoli of the ankle. The first slice at the level of the first cervical vertebra was manually contoured in full, after which the MIM software provided an estimation of the muscle contours for each subsequent centimetre. Each slice's contouring (every 1 cm) was meticulously reviewed by an experienced anatomist and edited as necessary to ensure accuracy. To exclude fat from the muscle mass, individual minimum and maximum grayscale thresholds were applied. These thresholds varied between individuals and also throughout different regions of the same individual. An illustrative processed MRI image is presented in Figure 1.

MIM software computed the muscle volume (cm^3^) based on the contouring and slice thickness (1 cm). Muscle mass (kg) was then calculated by multiplying the muscle volume by the density factor (1.04 g/cm^3^) [8, 26].

### 2.3 Ultrasound Assessment and Analysis

**FIGURE 2:**
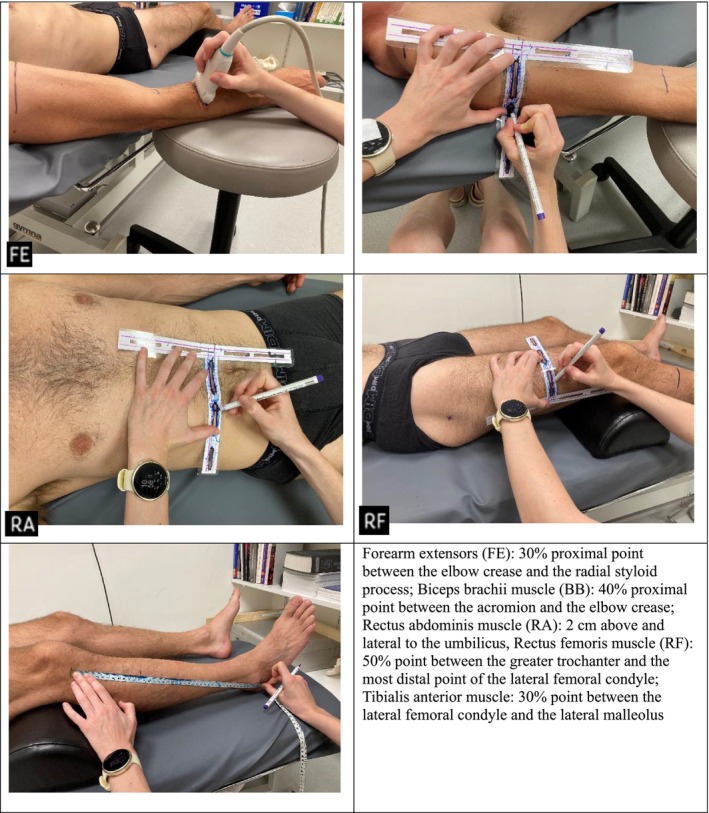
Anatomical landmarks and probe placement sites for ultrasound imaging of the assessed muscles in the supine position.

The ultrasound assessment began with the participant in the supine position, with the arms supported in 45° abduction. A 10‐cm‐thick pillow was placed under the knees to enhance comfort. Before performing ultrasound scanning, reference lines were meticulously applied to ensure accurate muscle measurements at the appropriate locations, as shown in Figure 2. Ultrasound measurements were conducted using a Mindray M7 equipped with a linear transducer (7L4s, 50 mm) and B‐mode extended‐field‐of‐view (EFOV) functionality. The ultrasound settings were configured as follows: depth 6.5 cm, frequency 10 MHz, dynamic range 65 dB, gain 60 dB, in‐phase sequence 1 and frame rate 93 fps. A generous amount of transmission gel was applied to ensure optimal image quality, and the transducer was positioned perpendicular to the skin surface. Panoramic ultrasound images were consistently captured from medial to lateral. To minimize proximal or distal probe drift during panoramic scanning, a drawn reference line on the skin was used as a guide for the probe. All scans were performed by an experienced researcher to ensure consistency and accuracy. Two scans were acquired for each muscle on the right side of the body, and average values were used for analysis.

**FIGURE 3:**
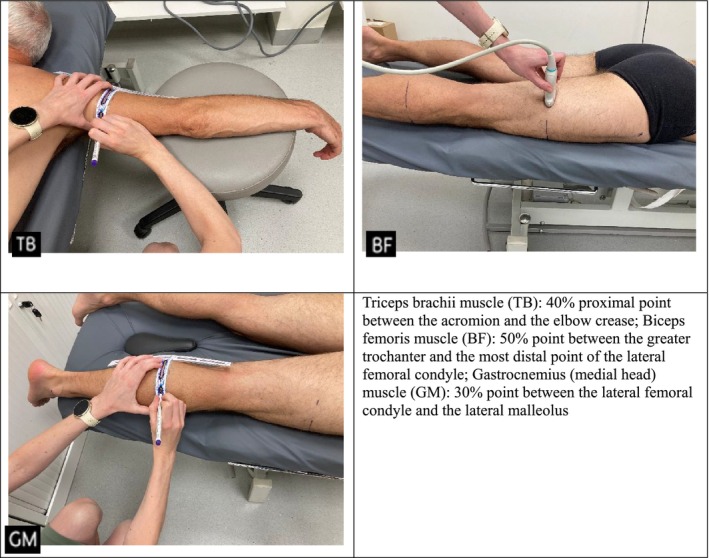
Extension of reference lines from anterior to posterior body surface using a set square.

For the second part of the ultrasound assessment, the participant was positioned in the prone position, with the feet hanging off the table and the arms supported in 90° abduction. The reference lines marked on the anterior side of the body were extended to the posterior side. To achieve this, a set square was used and aligned parallel to the limb, as illustrated in Figure 3. Measurement sites and muscle groups were selected based on established ultrasound protocols described in previous studies [8, 21, 27, 28], ensuring consistency with validated landmarks for MT and CSA assessment.

MT and CSA measurements were performed for the following muscles: biceps brachii, triceps brachii, rectus abdominis, rectus femoris, biceps femoris, tibialis anterior and gastrocnemius. Additionally, only MT measurements were obtained for the forearm extensor muscles. The ImageJ software [29] was employed for measurements.

**FIGURE 4:**
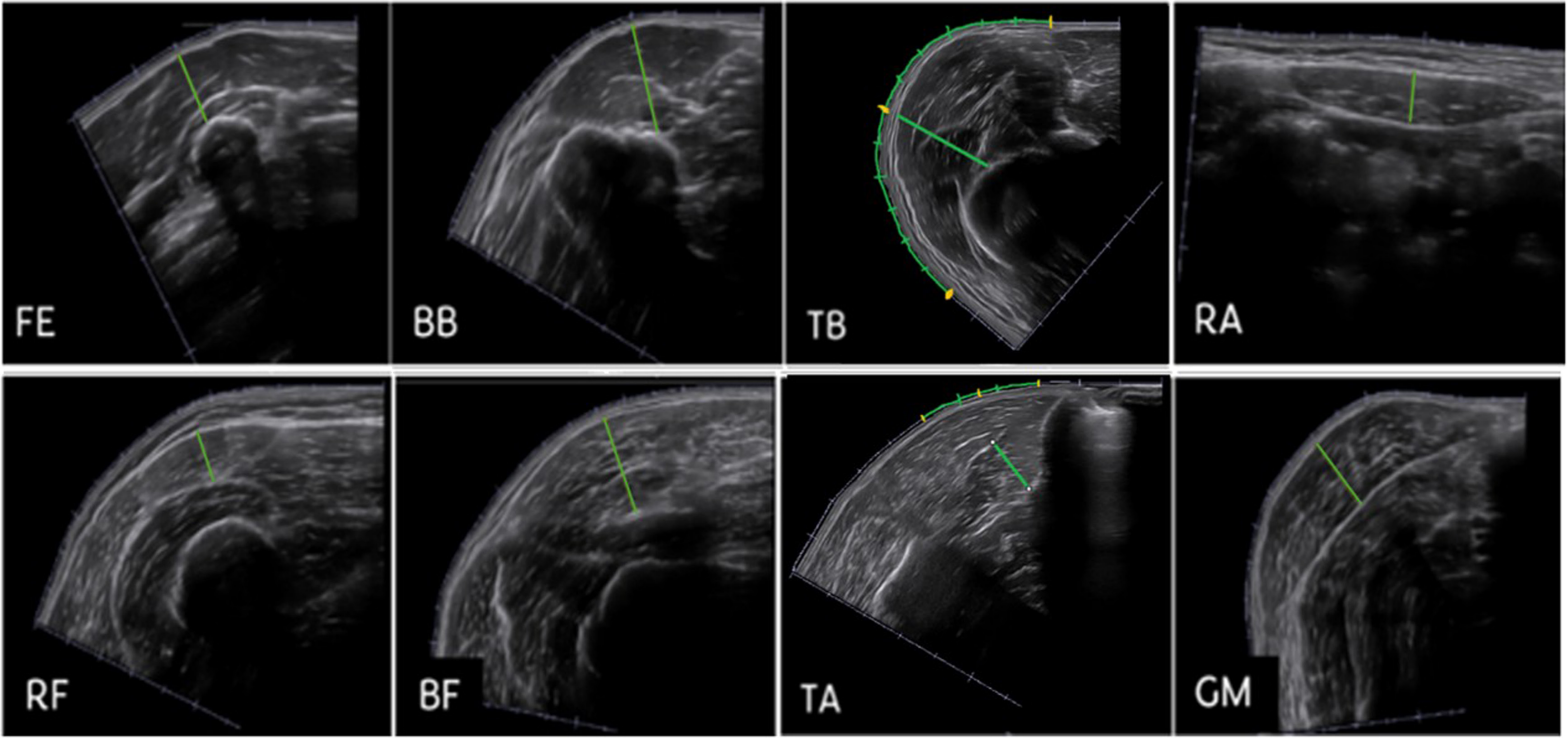
Muscle thickness measurements. BB = biceps brachii muscle, BF = biceps femoris muscle, FE = forearm extensor muscles, GM = gastrocnemius muscle (medial head), RA = rectus abdominis muscle, RF = rectus femoris muscle, TA = tibialis anterior muscle, TB = triceps brachii muscle.

Figure 4 illustrates an example of each MT measurement. For the forearm extensor muscles, MT was measured from the surface of the radius bone directly upward to the superficial fascia (i.e., distance between the underlying bone and the superficial fascia), with the measurement line placed perpendicular to the bone surface. For the biceps brachii, rectus abdominis, rectus femoris, biceps femoris and gastrocnemius muscles, thickness was measured between the superficial and deep muscle fascia. Measurement lines were positioned in such a way as to visually divide the muscle belly into two equal halves, ensuring consistency across participants. For the triceps brachii (combined long and medial heads) and tibialis anterior muscles, a different approach was taken due to their more complex morphology. For the triceps brachii muscle, the length of the visible muscle belly in the ultrasound image was first estimated using the centimetre calibration lines. MT was then measured at the midpoint of this visible muscle length, with the measurement lines placed perpendicular to the superficial fascia to the humerus. For the tibialis anterior muscle, the thickness was measured between the central aponeurosis of the muscle to the deep fascia, in such a way as to visually divide the muscle belly into two equal halves.

**FIGURE 5:**
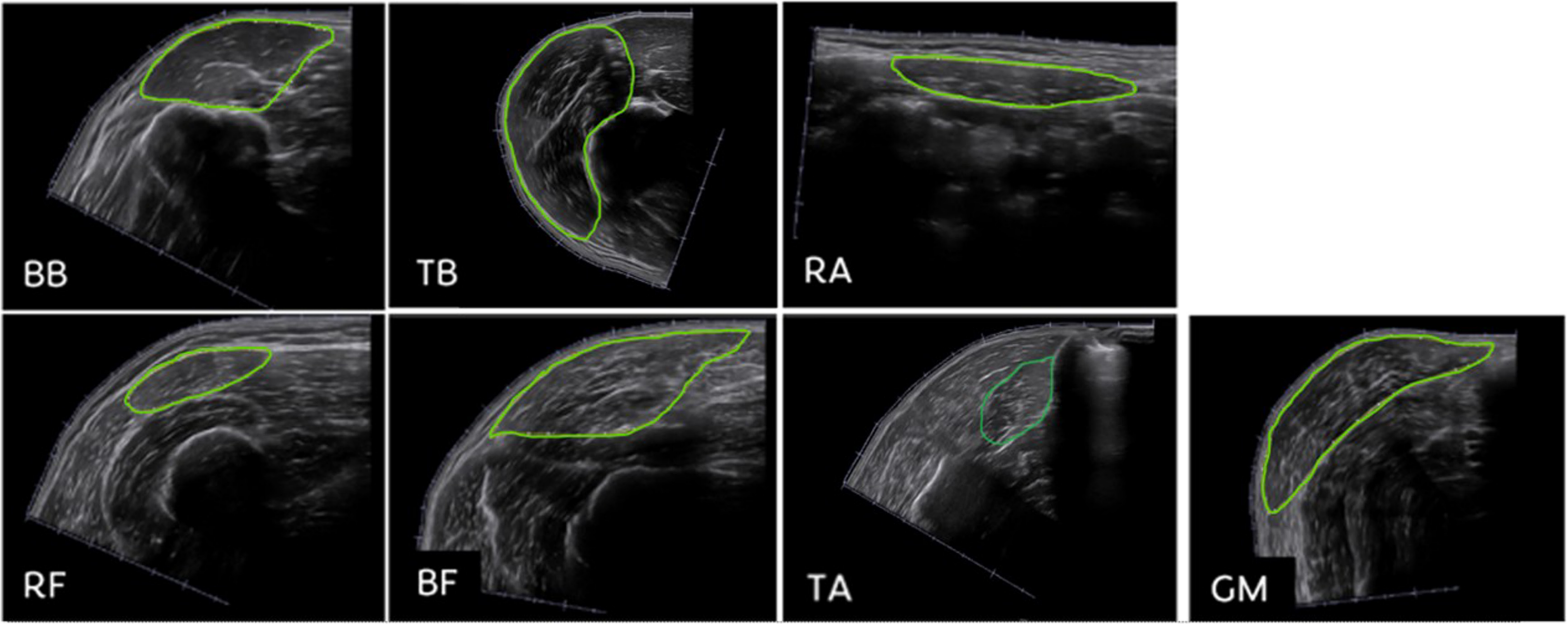
Cross‐sectional area measurements. BB = biceps brachii muscle, BF = biceps femoris muscle, GM = gastrocnemius muscle (medial head), RA = rectus abdominis muscle, RF = rectus femoris muscle, TA = tibialis anterior muscle, TB = triceps brachii muscle.

Figure 5 illustrates the methodology employed for drawing the CSA of each muscle on ImageJ. For CSA measurements, the muscle fascia was meticulously delineated. Notably, CSA measurements were not conducted for forearm extensor muscles due to the amalgamation of several extensors. The CSA of the biceps brachii and biceps femoris muscles encompassed both their long and short heads. In contrast, the CSA of the triceps brachii muscle included only the long and medial heads due to technical limitations with EFOV ultrasound [30]. If the lateral head were also included, overlap would occur on the ultrasound, and the medial head and the long head would no longer be fully visible. Similarly, the CSA of the gastrocnemius muscle solely accounted for its medial head.

Intra‐rater reliability of the ultrasound measurements was evaluated using the intra‐class correlation coefficient (ICC). The results indicated good to excellent intra‐rater reliability (ICC = 0.75–0.98), demonstrating high consistency and reproducibility of the ultrasound measurements within the same rater's assessments. This is consistent with previous ultrasound reliability research [13].

### 2.4 Statistical Analysis

G*Power (Version 3.1.9.4) was used to calculate the sample size for this study. For a multiple regression analysis, setting an effect size of 0.20, a significance level of 0.05, a power of 0.80, and considering 13 predictors (sex, age, height, weight, BMI + 8 MT measurements or 7 muscle CSA measurements), G*Power determined that a total of 101 participants were required. When considering 20 predictors (sex, age, height, weight, BMI + 8 MT measurements and 7 CSA measurements), 122 subjects are required.

To evaluate whether doubling right‐sided muscle mass provided an accurate estimate of WBMM within the MRI dataset, an additional comparison was performed in a subgroup of 93 participants for whom complete left‐ and right‐sided MRI data were available. WBMM was compared with the estimated value obtained by doubling right‐sided measurements. A Wilcoxon signed‐rank test was used to assess whether the differences between the values were statistically significant. A discrepancy smaller than 10% of the actual WBMM was considered acceptable.

For the allocation of participants into the development and cross‐validation groups, we employed a random selection method, assigning two‐thirds of the participants to the development group and the remaining one‐third to the validation group. The normality of the data distribution was assessed using the Shapiro–Wilk test. To explore potential differences between the development and cross‐validation groups, we used independent samples *t*‐tests for normally distributed numeric data, Mann–Whitney *U* tests for skewed numeric data and Fisher's exact test for nominal data.

In the development group of the study, three sets of multiple linear regression models were generated to estimate WBMM: one based on MT, one on CSA and one combining both MT and CSA. For each set, regression models were constructed using a forward stepwise selection method in SPSS. Variables were included based on statistical significance (*p* < 0.05), and models were evaluated based on the adjusted coefficient of determination (adjusted *R* ^2^) and the standard error of the estimate (SEE). Within each set, two equations were retained: one ‘optimized model’ with the best predictive performance (highest adjusted *R* ^2^ and lowest SEE) and one ‘practical model’ that required fewer variables. The practical model was selected by identifying the first solution in the stepwise procedure with an SEE ≤ 2.0 kg. This threshold was predefined, as 2.0 kg corresponds to < 10% of the median WBMM (~25 kg), and was considered the maximum acceptable prediction error. Multicollinearity was checked using tolerance values, and all included variables had tolerance values above 0.10.

The validity of the predicted muscle mass equations derived from the development group was evaluated against MRI‐measured muscle mass in the cross‐validation group. A Bland–Altman analysis was conducted to assess agreement between measured and predicted muscle mass values. This analysis provided mean differences (bias) and 95% limits of agreement (LoA) to quantify the degree of measurement error and potential systematic bias across the range of values. In addition to the Bland–Altman analysis, an ICC single‐measures analysis was performed to evaluate the agreement between MRI‐measured and ultrasound‐derived muscle mass measurements. In the ICC range of 0–0.20, reliability was considered slight; in the range of 0.21–0.50, poor; in the range of 0.51–0.75, moderate; in the range of 0.76–0.90, good; and > 0.91, excellent [31]. Following the ICC analysis, the standard error of measurement (SEM) was computed. The SEM was calculated by multiplying the standard deviation of the measurements by the square root of 1 minus the ICC value (SEM = SD × √(1 − ICC)). The coefficient of variation (CV%) was determined using the formula CV% = (SEM/mean) × 100. The combination of high ICC, low SEM and CV% and narrow limits of agreement with minimal bias was used to identify the most accurate predictive model. After cross‐validation, the equations were redeveloped using the full sample through multiple regression analysis.

All statistical analyses were performed using IBM SPSS Statistics Version 29.0.2.0, with a significance level set at *p* < 0.05.

## 3 Results

The comparison between measured WBMM and the value calculated by doubling the right‐sided muscle mass was performed using a Wilcoxon signed‐rank test. This test revealed a statistically significant difference (*p* = 0.01). However, the SEM was only 0.2 kg, indicating that despite statistical significance, the difference is not clinically relevant. Considering the median WBMM was 25.0 kg [19.1–30.1], this difference corresponds to less than 1% of the total muscle mass.

Table 1 presents the characteristics and muscle measurements of the participants, revealing no significant differences between the development and cross‐validation groups across all assessed variables.

**TABLE 1:** Characteristics and muscle measurements of the participants.

	Full sample (*N* = 211)	Development group (*N* = 141)	Cross‐validation group (*N* = 70)	*p*
Personal characteristics				
Sex (m/f)	102/109	68/73	34/36	1.000^a^
Age (y)	42.0 [29.0–58.0]	42.0 [30.0–57.5]	41.0 [28.0–58.0]	0.629^b^
Height (m)	1.71 ± 0.10	1.71 ± 0.10	1.72 ± 0.10	0.590^c^
Weight (kg)	69.0 [62.0–80.0]	70.7 ± 12.5	72.1 ± 13.0	0.862^c^
BMI (kg/m^2^)	24.1 ± 3.4	24.1 ± 3.2	24.2 ± 3.6	0.318^c^
MRI muscle mass measurement				
Whole‐body muscle mass (kg)	25.0 [19.1–30.1]	24.2 [19.0–29.9]	25.2 [19.8–31.2]	0.244^b^
Muscle thickness measurements				
Forearm extensors (cm)	1.6 ± 0.4	1.6 ± 0.3	1.6 ± 0.4	0.303^c^
Biceps brachii (cm)	2.1 ± 0.5	2.1 [1.8–2.4]	2.1 ± 0.5	0.953^b^
Triceps brachii (cm)	2.5 [2.1–2.9]	2.5 [2.1–2.8]	2.6 ± 0.6	0.386^b^
Rectus abdominis (cm)	1.0 [0.8–1.2]	1.0 [0.8–1.2]	1.1 ± 0.3	0.471^b^
Rectus femoris (cm)	1.3 ± 0.3	1.3 ± 0.3	1.3 ± 0.3	0.073^c^
Biceps femoris (cm)	2.8 [2.5–3.1]	2.8 ± 0.5	2.8 [2.6–3.2]	0.254^b^
Tibialis anterior (cm)	2.4 ± 0.3	2.4 ± 0.3	2.3 ± 0.3	0.622^c^
Gastrocnemius (cm)	1.6 ± 0.3	1.6 ± 0.3	1.6 ± 0.3	0.562^c^
Muscle cross‐sectional area measurements				
Biceps brachii (cm^2^)	6.0 [4.8–7.8]	5.9 [4.6–7.7]	6.2 [5.1–8.4]	0.273^b^
Triceps brachii (cm^2^)	12.5 [10.1–16.0]	12.0 [10.0–15.9]	13.1 [10.1–16.1]	0.317^b^
Rectus abdominis (cm^2^)	5.5 [4.1–6.7]	5.5 [4.1–6.5]	5.4 [4.1–7.1]	0.936^b^
Rectus femoris (cm^2^)	4.2 [3.3–5.3]	4.2 [3.5–5.3]	4.3 ± 1.6	0.603^b^
Biceps femoris (cm^2^)	10.4 [8.7–12.3]	10.4 [8.6–12.4]	10.5 [9.1–12.2]	0.707^b^
Tibialis anterior (cm^2^)	2.4 [2.0–3.5]	2.3 [2.0–3.1]	2.6 [2.0–4.2]	0.164^b^
Gastrocnemius (cm^2^)	9.5 [7.8–11.2]	9.3 [7.7–10.9]	10.0 ± 2.8	0.329^b^

Abbreviations: f = female, m = male, MRI = magnetic resonance imaging.

^a^ Fisher's exact test.

^b^ Mann–Whitney *U* test.

^c^ Independent samples *t*‐test.

Table 2 shows the prediction equations for WBMM, based on the development group. Sex is a constant variable included in each equation. Additionally, ultrasound measurements of the rectus abdominis, biceps femoris and tibialis anterior muscles are constants in all equations.

**TABLE 2:** Prediction equations for whole‐body muscle mass, based on the development group (*N* = 141).

Equations based on muscle thickness measurements		*R* ^2^	Adjusted *R* ^2^	SEE (kg)	*p*
Model 1	WBMM = −2.21 + (2.17 × FEMT) + (1.36 × TBMT) + (4.08 × RAMT) + (1.77 × RFMT) + (1.69 × BFMT) + (3.06 × TAMT) − (3.68 × sex) + (0.23 × weight) − (0.53 × BMI)	0.945	0.941	1.7	< 0.001
Model 2	WBMM = −27.75 + (6.80 × RAMT) + (2.2 × BFMT) + (4.12 × TAMT) − (4.72 × sex) + (18.78 × height)	0.917	0.914	2.0	< 0.001

Equations based on CSA measurements		*R* ^2^	Adjusted *R* ^2^	SEE (kg)	*p*
Model 3	WBMM = −12.87 − (0.04 × age) + (0.25 × TBCSA) + (0.69 × RACSA) + (0.28 × BFCSA) + (0.89 × TACSA) + (0.19 × GMCSA) − (4.63 × sex) + (16.10 × height)	0.940	0.936	1.8	< 0.001
Model 4	WBMM = −16.93 + (0.32 × TBCSA) + (0.73 × RACSA) + (0.34 × BFCSA) + (1.06 × TACSA) − (4.12 × sex) + (17.03 × height)	0.929	0.926	1.9	< 0.001

Equations based on muscle thickness and CSA measurements		*R* ^2^	Adjusted *R* ^2^	SEE (kg)	*p*
Model 5	WBMM = 1.54 + (1.97 × FEMT) + (3.46 × RAMT) + (1.63 × RFMT) + (1.60 × BFMT) + (2.03 × TAMT) + (0.24 × TBCSA) + (0.44 × TACSA) − (3.65 × sex) + (0.23 × weight) − (0.55 × BMI)	0.951	0.948	1.6	< 0.001
Model 6	WBMM = −28.42 + (4.29 × RAMT) + (2.16 × BFMT) + (3.81 × TAMT) + (0.31 × TBCSA) − (3.76 × sex) + (18.49 × height)	0.935	0.932	1.8	< 0.001

*Note:* Sex: male = 0; female = 1, weight (kg), BMI (kg/m^2^), height (m), age (y).

Abbreviations: BFCSA = biceps femoris CSA (cm^2^), BFMT = biceps femoris MT (cm), CSA = cross‐sectional area, FEMT = forearm extensors MT (cm), GMCSA = gastrocnemius CSA (cm^2^), MT = muscle thickness, RACSA = rectus abdominis CSA (cm^2^), RAMT = rectus abdominis MT (cm), RFMT = rectus femoris MT (cm), SEE = standard error of the estimate, TACSA = tibialis anterior CSA (cm^2^), TAMT = tibialis anterior MT (cm), TBCSA = triceps brachii CSA (cm^2^), TBMT = triceps brachii MT (cm), WBMM = whole‐body muscle mass (kg).

Agreement between predicted and measured muscle mass was assessed for each of the six regression models using both Bland–Altman analysis and reliability analysis, as shown in Table 3. Model 1, based on muscle thickness with the lowest SEE, showed excellent agreement with a mean bias of 0.79 kg (SD = 1.99) and LoA ranging from −3.11 to 4.69 kg. The ICC was 0.97, indicating excellent reliability. Model 2, which applied a maximum SEE of 2 kg using muscle thickness, showed a slightly larger bias of 0.97 kg (SD = 2.29; LoA: −3.51–5.46 kg) and an ICC of 0.95. Model 3, based on CSA with the lowest SEE, resulted in a lower bias (0.29 kg), but a wider range of agreement (LoA: −4.57–5.15 kg; SD = 2.48), and an ICC of 0.95. Model 4, which limited SEE to 2 kg based on CSA, showed similar agreement (bias = 0.24 kg, SD = 2.60; LoA: −4.86–5.34 kg; ICC = 0.94). Model 5, which combined muscle thickness and CSA with the lowest SEE, demonstrated the smallest bias of all models (−0.03 kg), narrow LoA (−3.99–3.92 kg) and an ICC of 0.97—indicating both high agreement and minimal systematic error. Model 6, based on combined measurements with an SEE capped at 2 kg, also showed strong performance (bias = 0.02 kg, SD = 2.24; LoA: −4.37–4.41 kg), with an ICC of 0.96. The representative Bland–Altman plots of these models are available as Supporting Information. Visual inspection of the Bland–Altman plots revealed no clear trend across the range of muscle mass values, suggesting that the prediction errors were homogeneously distributed across both low and high muscle mass participants.

**TABLE 3:** Cross‐validation of the ultrasound‐derived equations (*N* = 70).

	MRI measured muscle mass (kg)	Ultrasound calculated muscle mass (kg)	Bland–Altman			Reliability analysis			
			Bias (kg)	SD (kg)	95% LoA (kg)	ICC	*p*	SEM (kg)	CV (%)
Equations based on muscle thickness measurements									
Model 1	25.2 [19.8–31.2]	25.1 [19.2–30.2]	0.79	1.99	−3.11;4.69	0.965	< 0.001	1.4	5.5
Model 2		24.8 [18.5–30.2]	0.97	2.29	−3.51;5.46	0.953	< 0.001	1.7	6.4
Equations based on CSA measurements									
Model 3	25.2 [19.8–31.2]	25.6 [19.4–30.4]	0.29	2.48	−4.57;5.15	0.945	< 0.001	1.8	6.9
Model 4		25.9 [19.0–30.6]	0.24	2.60	−4.86;5.34	0.940	< 0.001	1.9	7.2
Equations based on muscle thickness and CSA measurements									
Model 5	25.2 [19.8–31.2]	25.9 [19.9–30.8]	−0.03	2.02	−3.99;3.92	0.965	< 0.001	1.4	5.4
Model 6		26.4 [19.6–30.6]	0.02	2.24	−4.37;4.41	0.956	< 0.001	1.6	6.1

*Note:* MRI measured muscle mass (median [interquartile range]), ultrasound calculated muscle mass (median [interquartile range]).

Abbreviations: CV = coefficient of variation, ICC = intraclass correlation coefficient, LoA = limits of agreement, MRI = magnetic resonance imaging, SD = standard deviation, SEM = standard error of measurement.

Because the adj. *R* ^2^ and SEE of the equations based on the combination of muscle thickness and CSA measurements scored best, we applied both approaches to the full sample in Table 4. The most accurate equation consists of 10 variables: sex, weight, BMI and seven ultrasound muscle measurements. For the muscle thickness measurements, two arm muscles (forearm extensor and triceps brachii muscles), one abdominal muscle (rectus abdominis muscle) and three leg muscles (rectus femoris, biceps femoris and tibialis anterior muscles) were included. One muscle CSA measurement of the triceps brachii muscle was used.

**TABLE 4:** Prediction equations for whole‐body muscle mass, based on the full sample (*N* = 211).

Declaration of variables	*R* ^2^	Adjusted *R* ^2^	SEE (kg)
Equations based on muscle thickness measurements and CSA measurements			
WBMM = 0.302 + (2.21 × FEMT) + (3.73 × RAMT) + (1.39 × RFMT) + (1.84 × BFMT) + (2.04 × TAMT) + (0.22 × TBCSA) + (0.27 × TACSA) − (3.29 × sex) + (0.25 × weight) − (0.60 × BMI)	0.945	0.942	1.7
WBMM = −31.69 + (4.21 × RAMT) + (2.31 × BFMT) + (3.56 × TAMT) + (0.33 × TBCSA) − (3.42 × sex) + (20.38 × height)	0.929	0.927	2.0

*Note:* Sex: male = 1; female = 0, weight (kg).

Abbreviations: BFMT = biceps femoris MT (cm), BMI = body mass index (kg/m^2^), CSA = cross‐sectional area, FEMT = forearm extensors MT (cm), MT = muscle thickness, RAMT = rectus abdominis MT (cm), RFMT = rectus femoris MT (cm), SEE = standard error of the estimate, TACSA = tibialis anterior CSA (cm^2^), TAMT = tibialis anterior MT (cm), TBCSA = triceps brachii CSA (cm^2^), WBMM = whole‐body muscle mass.

The most practical equation consists of six variables: sex, height and four ultrasound muscle measurements. For the muscle measurements, one arm muscle (triceps brachii muscle CSA), one abdominal muscle (rectus abdominis muscle) and two leg muscles (biceps femoris and tibialis anterior muscle) were used.

## 4 Discussion

The objective of this study was to develop equations for predicting WBMM using muscle ultrasound measurements.

The muscles included in the equations of this study differ from the ultrasound measurements used in previous research. Earlier studies in the literature often used muscle thickness measurements at specific body sites [8, 21, 22, 27, 28, 32], whereas this study focused on the thickness of individual muscles. This choice was made because measuring individual muscles is more accurate [33], as each muscle undergoes age‐related atrophy differently [34]. In addition to using muscle thickness ultrasound measurements, this is the first study to include CSA measurements alongside individual muscle thickness in predictive equations. The addition of CSA measurements—specifically of the triceps brachii and tibialis anterior—to models based on muscle thickness resulted in improved accuracy, as reflected by higher *R* ^2^ values and lower SEE. These findings suggest that combining CSA and thickness measurements may enhance prediction performance and should be considered in future practice.

When comparing the equations from this study with those developed in previous literature, it becomes immediately apparent that sex plays a significant role in WBMM [12, 27, 28, 32, 35]. Whereas some researchers also included ‘sex’ as a variable in their equations, others created separate equations for men and women [8, 21].

Whether height, weight and BMI play a significant role is less clear from this study. All these variables were included in the equations, but their use varied depending on the accuracy and the specific muscle measurements used. The literature also lacks consensus on this matter. Although all these variables appear in various equations, there is no agreement on which is the most important or influential. In the most accurate equation from this study, weight and BMI are combined, suggesting that weight may overestimate muscle mass and is corrected by BMI. In contrast, the most practical equation includes height, which shows a strong correlation with WBMM [8, 12, 21, 27, 32, 35, 36].

The variable ‘age’ appears less frequently in equations from prior research [27, 36], indicating that ‘age’ contributes little additional value to the equation. This finding is confirmed in the present study, where age does not play a role in the equations for estimating muscle mass.

The prevalent approach in prior studies involves conducting ultrasound muscle measurements with participants in a standing position [27, 28, 35]. Conversely, in analogous investigations, ultrasound muscle measurements were executed with participants in a lying position [12, 32]. The utility of a lying position has been validated for its enhanced reliability and practical feasibility [37]. Notably, muscle relaxation is compromised in a standing position, a factor mitigated in MRI examinations where participants are typically examined in a lying position to minimize measurement artefacts.

The strength of this study lies in the cross‐validation of both equations. The most accurate equation uses 10 variables to estimate WBMM, with a potential error of 1.7 kg. Other equations have larger errors or lack cross‐validation [8, 12, 22, 28, 36, 38], making them less reliably accurate. On the other hand, the more practical equation has a slightly larger potential error (2.0 kg), but it was also cross‐validated, in contrast to previous research [12, 27, 28, 32, 35], and still demonstrates a small margin of error. In earlier studies, equations using four or fewer variables were also developed, but these had a larger margin of error [22].

An important methodological consideration is the standardized placement of measurement lines for muscle thickness, particularly for muscles with more complex or variable morphology. Whereas muscles such as the rectus femoris and rectus abdominis present with a well‐defined muscle belly, other muscles—such as the triceps brachii and tibialis anterior—require more careful consideration. For the triceps brachii muscle, only the long and medial heads were included in the CSA assessment, whereas for the tibialis anterior muscle, only the deep portion of the muscle was measured. The aponeurosis of the deep part of the tibialis anterior is clearly visible on ultrasound, which, in our experience, allows for more accurate and reliable delineation of the muscle boundaries. In this study, consistent internal protocols were followed for measurement line placement, with careful training and verification to ensure reproducibility. However, we acknowledge that such measurements may be prone to inter‐rater variation, especially when the anatomical landmarks are less clearly defined. Future studies should focus on further refining and validating standardized protocols for these muscle sites, potentially by comparing different measurement strategies (e.g., bone‐to‐fascia vs. fascia‐to‐fascia) across populations and devices.

Although posterior measurements require participant repositioning, their inclusion proved necessary to maintain prediction accuracy. Models based on anterior landmarks only (e.g., forearm extensor, biceps brachii, rectus abdominis, rectus femoris and tibialis anterior muscles) yielded substantially higher standard errors. These findings suggest that a compromise in practicality leads to reductions in accuracy. To explore the feasibility of using only anterior landmarks for practical applications, we conducted an additional series of stepwise regression analyses based on anterior sites only (see Table S1).

An important avenue for future research lies in the development of limb‐specific prediction equations. Although the current study focused on WBMM estimation, localized assessments—such as separate predictions for the quadriceps, hamstrings or trunk musculature—may provide added clinical and functional relevance. Such region‐specific models could be particularly valuable in populations where muscle loss is asymmetrical or segmental (e.g., orthopaedic injury, stroke or sarcopenia). The current dataset offers a solid foundation for developing and validating these localized models. Future work will aim to explore these possibilities in order to broaden the applicability of ultrasound‐based muscle mass assessment.

To determine low muscle mass, a skeletal muscle index (SMI) is often used in practice, where WBMM is divided by height squared. Considering the average height of the participants in this study (1.71 m), the potential error in the SMI would be 0.7 kg/m^2^ (2.0 kg/(1.71 m)^2^). This should be taken into account when using the ultrasound‐derived equations in practice for diagnosing low muscle mass.

An acknowledged constraint of MRI is its inability to accommodate individuals with wider body frames, a limitation observed in our study as well. To mitigate this, we adopted a unilateral measurement approach, doubling the recorded values to estimate WBMM. Although we observed a significant difference between MRI‐measured and ultrasound‐based calculated muscle mass, the small SEM of 0.2 kg suggests negligible practical significance. Thus, we assert that this unilateral measurement strategy remains sufficiently reliable for estimating WBMM.

This investigation was conducted on a Caucasian sample, which, in previous literature, has only been explored using DXA as the reference method [12, 35]. Previous MRI‐based studies were limited to small Asian samples of 61 and 72 participants [8, 21]. In contrast, our study included 211 participants, meeting the sample size requirements. Because body composition differs between ethnicities [22], and our research was only conducted in healthy Caucasians, the developed equation can only be used in this population. Further research is needed to determine whether this equation is also useful in a sample where the participants are not healthy. Our study focused on predicting WBMM, which contrasts with the predominant approach in previous literature, where equations were developed primarily to estimate appendicular lean mass [12, 27, 32, 35, 36]. Consequently, these equations did not incorporate muscle mass from the trunk [19], potentially presenting a limitation. This is important because age‐related muscle loss is not confined solely to the legs but also affects the trunk [8, 28, 39]. Conversely, the decline in muscle mass in the arms exhibits less correlation with age [8, 28].

## 5 Conclusion

The proposed equations from this study enable the accurate prediction of individual WBMM in a Caucasian population based on muscle measurements using ultrasound. There can be opted for a more practical equation, which requires only four ultrasound measurements, or there can be chosen for a more accurate equation, which does require a greater time investment but offers a more accurate result. This study shows that the addition of CSA measurements has influence on the accuracy of the equations. Therefore, in the future, it is recommended to use the CSA measurements to improve the accuracy of WBMM prediction.

Looking ahead, it is imperative to assess the reliability and applicability of these equations in non‐healthy samples to ensure their suitability for clinical practice. By further validating these equations in diverse populations and clinical settings, we can enhance their utility in diagnosing and monitoring muscle‐related conditions, ultimately improving healthcare outcomes for individuals worldwide.

## Funding

The authors have nothing to report.

## Ethics Statement

The study protocol received approval from the ethics committee (B.U.N. 1432020000335).

## Conflicts of Interest

The authors declare no conflicts of interest.

## Supporting information


**Data S1:** Supporting Information.
