# Supplementary material for: Novel MRI‐Guided Ultrasound Equations for Whole‐Body Muscle Mass in Caucasian Adults
Source: J Cachexia Sarcopenia Muscle. 2026 Mar 19;17(2):e70242. doi: 10.1002/jcsm.70242 (PMC13140327; doi:10.1002/jcsm.70242)
Supplement: Supplementary file 1 — Data S1: Supporting Information. [file JCSM-17-e70242-s001.docx]

Supplementary material

Bland-Altman plots

Model 1: WBMM = - 2.21 + (2.17 * FEMT) + (1.36 * TBMT) + (4.08 * RAMT) + (1.77 * RFMT) + (1.69 * BFMT) + (3.06 * TAMT) – (3.68 * sex) + (0.23 * weight) – (0.53 * BMI)
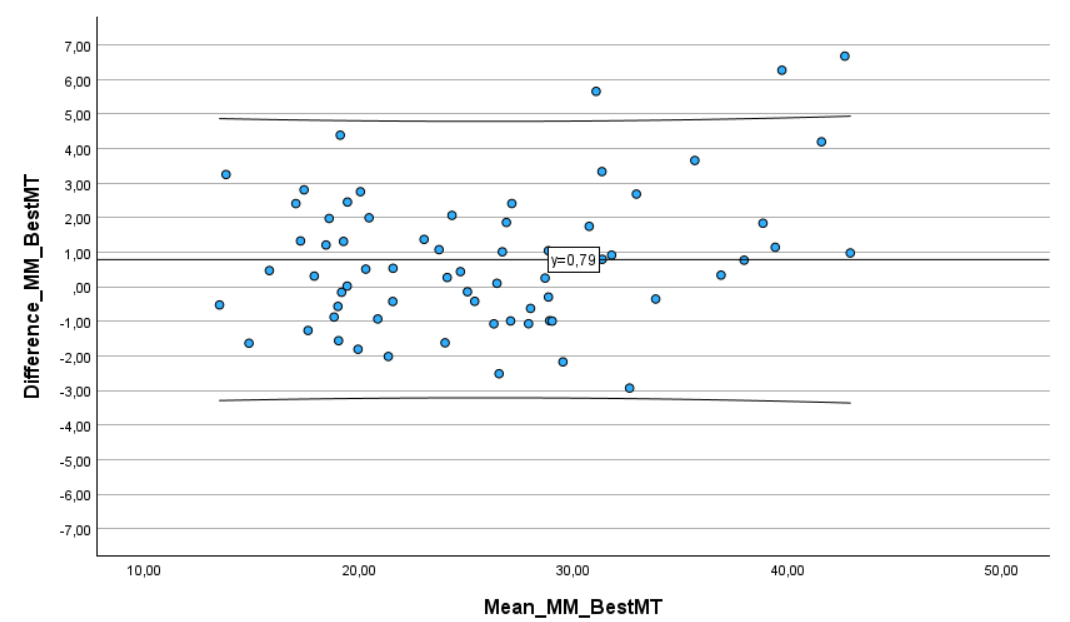

WBMM=Whole-body muscle mass (kg), FEMT=Forearm extensor muscle thickness (cm), TBMT=Triceps brachii muscle thickness (cm), RAMT=Rectus abdominis muscle thickness (cm), RFMT=Rectus femoris muscle thickness (cm), BFMT=Biceps femoris muscle thickness (cm), TAMT= Tibialis anterior muscle thickness (cm), sex (male=0, female=1), weight (kg), BMI=Body mass index (kg/m²), MM=Muscle mass (kg)

Model 2: WBMM = - 27.75 + ( 6.80 * RAMT) + (2.21* BFMT) + (4.12* TAMT) – (4.72 * sex) + (18.78 * height)

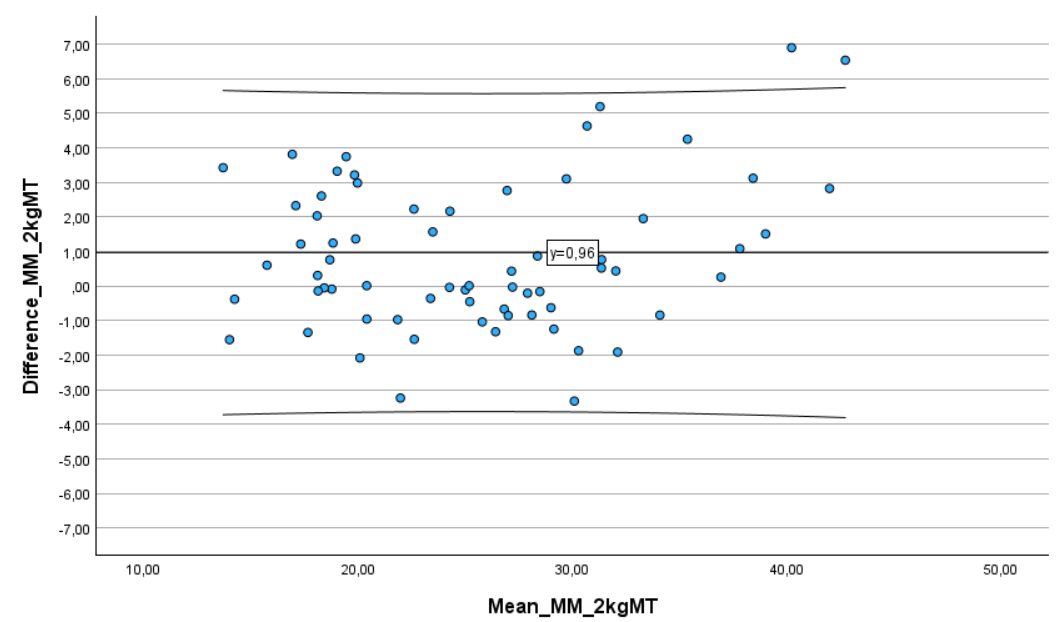
WBMM=Whole-body muscle mass (kg), RAMT=Rectus abdominis muscle thickness (cm), BFMT= Biceps femoris muscle thickness (cm), TAMT= Tibialis anterior muscle thickness (cm), sex (male=0, female=1), height (m), MM=Muscle mass (kg)

Model 3: WBMM = - 12.87 – (0.04 * age) + (0.25 * TBCSA) + (0.69 * RACSA) + (0.28 * BFCSA) + (0.89 * TACSA) + (0.19 * GMCSA) – (4.63 * sex) + (16.10 * height)
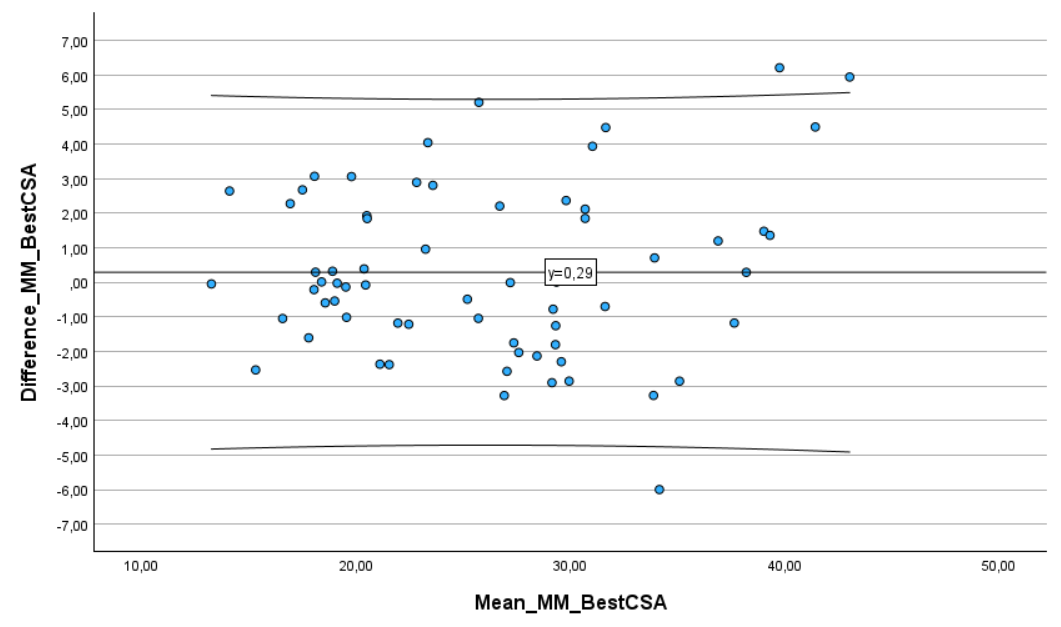

WBMM=Whole-body muscle mass (kg), TBCSA=Triceps brachii cross-sectional area (cm²), RACSA=Rectus abdominis cross-sectional area (cm²), BFCSA=Biceps femoris cross-sectional area (cm²), TACSA=Tibialis anterior cross-sectional area (cm²), GMCSA=Gastrocnemius (medial head) cross-sectional area (cm²), sex (male=0, female=1), height (m), MM=Muscle mass (kg)

Model 4: WBMM = - 16.93 + (0.32 * TBCSA) + (0.73 * RACSA) + (0.34 * BFCSA) + (1.06 * TACSA) – (4.12 * sex) + (17.03 * height)
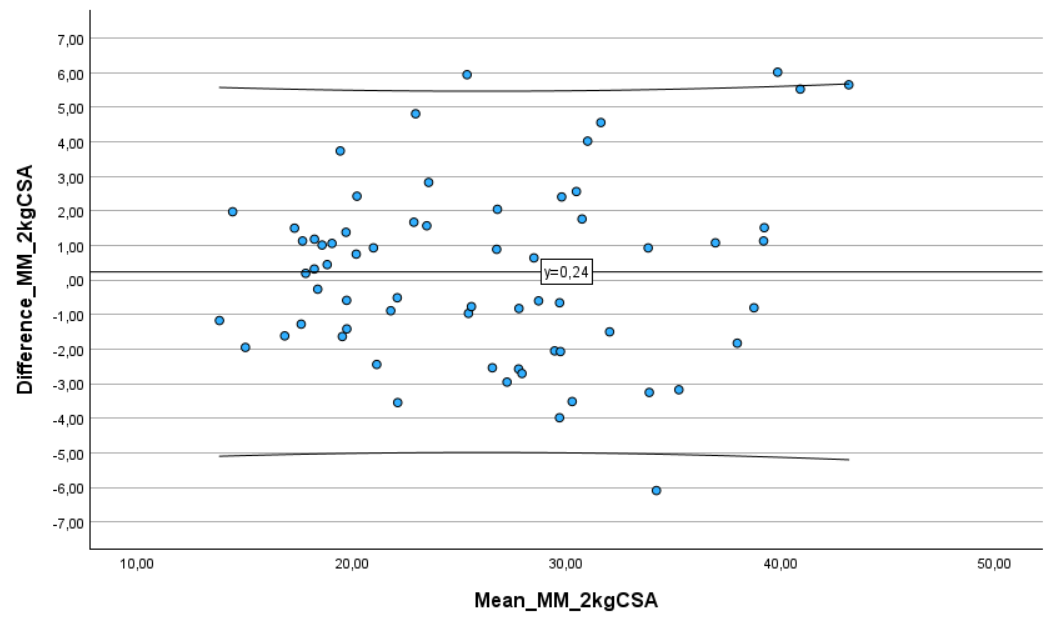

WBMM=Whole-body muscle mass (kg), TBCSA=Triceps brachii cross-sectional area (cm²), RACSA=Rectus abdominis cross-sectional area (cm²), BFCSA=Biceps femoris cross-sectional area (cm²), TACSA=Tibialis anterior cross-sectional area (cm²), sex (male=0, female=1), height (m), MM=Muscle mass (kg)

Model 5: WBMM = 1.54 + (1.97 * FEMT) + (3.46 * RAMT) + (1.63 * RFMT) + (1.60 * BFMT) + (2.03 * TAMT) + (0.24 * TBCSA) + (0.44 * TACSA) – (3.65 * sex) + (0.23 * weight) – (0.55 * BMI)
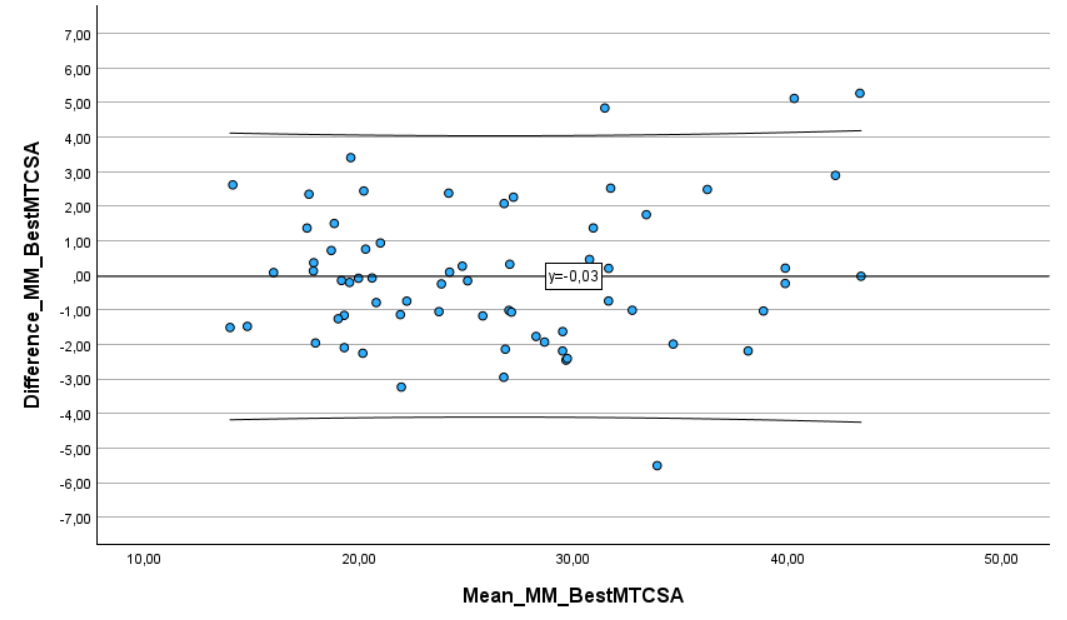

WBMM=Whole-body muscle mass (kg), FEMT=Forearm extensor muscle thickness (cm), RAMT=Rectus abdominis muscle thickness (cm), RFMT=Rectus femoris muscle thickness (cm), BFMT=Biceps femoris muscle thickness (cm), TAMT=Tibialis anterior muscle thickness (cm), TBCSA=Triceps brachii cross-sectional area (cm²), TACSA=Tibialis anterior cross-sectional area (cm²), sex (male=0, female=1), weight (kg), BMI=Body mass index (kg/m²), MM=Muscle mass (kg)

Model 6: WBMM = - 28.42 + (4.29 * RAMT) + (2.16 * BFMT) + (3.81 * TAMT) + (0.31 * TBCSA) – (3.76 * sex) + (18.49 * height)
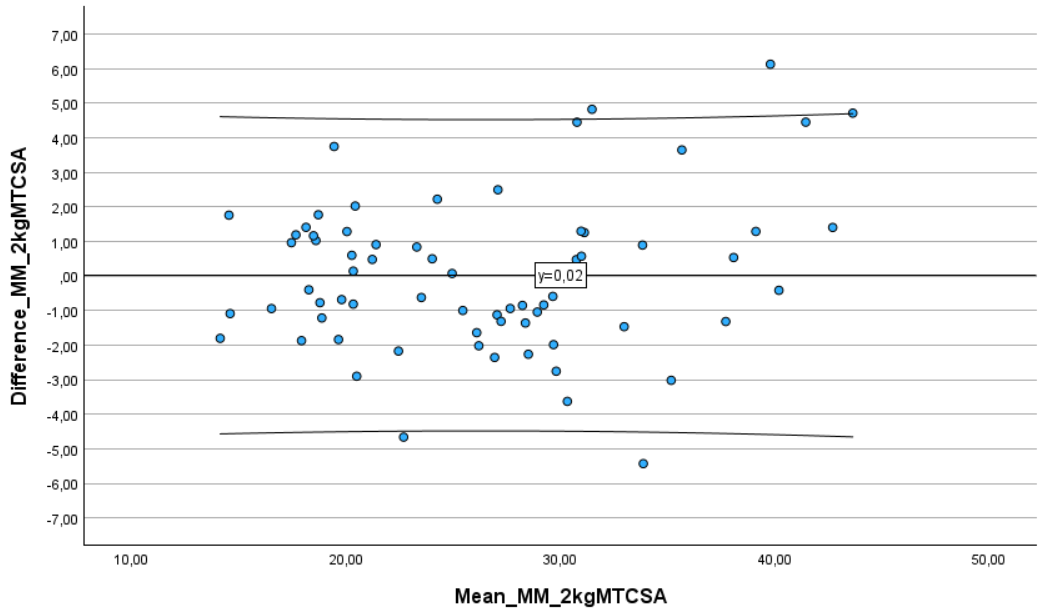

WBMM=Whole-body muscle mass (kg), RAMT=Rectus abdominis muscle thickness (cm), BFMT=Biceps femoris muscle thickness (cm), TAMT=Tibialis anterior muscle thickness (cm), TBCSA=Triceps brachii cross-sectional area (cm²), sex (male=0, female=1), height (m), MM=Muscle mass (kg)

Table A. Prediction equations for whole-body muscle mass, based on only anterior landmarks (N=211)

| Equations based on muscle thickness measurements | R² | Adjusted R² | SEE (kg) | p-value |
| --- | --- | --- | --- | --- |
| WBMM = 3.57 + (5.09 * RAMT) + (2.93 * TAMT) + (3.12 * FEMT) + (1.45 * BBMT) + (0.25 * weight) – (3.87 * sex) – (0.05 * age) – (0.51 * BMI) | 0.929 | 0.926 | 1.9 | <0.001 |
| Equations based on CSA measurements | R² | Adjusted R² | SEE (kg) |  |
| WBMM = - 17.30 + (0.67 * BBCSA) + (0.89 * TACSA) + (0.8 * RAMT) + (0.29 * RFMT) + (18.00 * height) + (0.06 * weight) – (3.88 * sex) – (0.05 * age) | 0.915 | 0.912 | 2.1 | <0.001 |
| Equations based on muscle thickness and CSA measurements | R² | Adjusted R² | SEE (kg) |  |
| WBMM = 4.42 + (4.49 * RAMT) + (1.91 * TAMT) + (2.67 * FEMT) + (1.32 * RFMT) + (0.42 * BBCSA) + (0.45 * TACSA) + (0.26 * weight) – (3.50 * sex) – (0.57 * BMI) – (0.03 * age) | 0.934 | 0.931 | 1.9 | <0.001 |

WBMM=whole-body muscle mass, SEE=standard error of the estimate, MT= Muscle thickness, CSA=Cross-sectional area, FEMT= Forearm extensors MT (cm), RAMT= Rectus abdominis MT (cm), RFMT= Rectus femoris MT (cm), TAMT= Tibialis anterior MT (cm), Sex: male=1; female=0, Weight (kg), BMI (kg/m²), Height (m), Age (y), RACSA = Rectus abdominis CSA (cm²), TACSA= Tibialis anterior CSA, (cm²)
